# Na/K-ATPase Gene Expression in the Human Cochlea: A Study Using mRNA *in situ* Hybridization and Super-Resolution Structured Illumination Microscopy

**DOI:** 10.3389/fnmol.2022.857216

**Published:** 2022-03-31

**Authors:** Wei Liu, Helge Rask-Andersen

**Affiliations:** Section of Otolaryngology, Department of Surgical Sciences, Head and Neck Surgery, Uppsala University Hospital, Uppsala, Sweden

**Keywords:** human, inner ear, Na/K-ATPase gene, RNAscope, structured illumination microscopy

## Abstract

**Background:**

The pervasive Na/K-ATPase pump is highly expressed in the human cochlea and is involved in the generation of the endocochlear potential as well as auditory nerve signaling and relay. Its distribution, molecular organization and gene regulation are essential to establish to better understand inner ear function and disease. Here, we analyzed the expression and distribution of the *ATP1A1*, *ATP1B1*, and *ATP1A3* gene transcripts encoding the Na/K-ATPase α1, α3, and β1 isoforms in different domains of the human cochlea using RNA *in situ* hybridization.

**Materials and Methods:**

Archival paraformaldehyde-fixed sections derived from surgically obtained human cochleae were used to label single mRNA gene transcripts using the highly sensitive multiplex RNAscope^®^ technique. Localization of gene transcripts was performed by super-resolution structured illumination microscopy (SR-SIM) using fluorescent-tagged probes. *GJB6* encoding of the protein connexin30 served as an additional control.

**Results:**

Single mRNA gene transcripts were seen as brightly stained puncta. Positive and negative controls verified the specificity of the labeling. *ATP1A1* and *ATP1B1* gene transcripts were demonstrated in the organ of Corti, including the hair and supporting cells. In the stria vascularis, these transcripts were solely expressed in the marginal cells. A large number of *ATP1B1* gene transcripts were found in the spiral ganglion cell soma, outer sulcus, root cells, and type II fibrocytes. The *ATP1B1* and *ATP1A3* gene transcripts were rarely detected in axons.

**Discussion:**

Surgically obtained inner ear tissue can be used to identify single mRNA gene transcripts using high-resolution fluorescence microscopy after prompt formaldehyde fixation and chelate decalcification. A large number of Na/K-ATPase gene transcripts were localized in selected areas of the cochlear wall epithelium, fibrocyte networks, and spiral ganglion, confirming the enzyme’s essential role for human cochlear function.

## Introduction

The human cochlea contains complex cell assemblies which transform sound into electrical nerve signals to the brain. The hearing organ contains 15,000 sensory hair cells which transduce mechanical energy, but only a fraction (3,400) convey direct signals to the 30,000 nerve fibers (Retzius, 1884). The receptors are supported by eight different types of specialized epithelial cells. Their apical poles are submerged in a K^+^-rich endolymph fluid whose electrochemical feature plays a key role in receptor function. A field or endocochlear potential (EP) is generated in the lateral wall of the cochlea that contains a unique vascularized, tri-cellular epithelium named stria vascularis (SV), which is linked to a series of mesenchymal cells called fibrocytes (Smith et al., 1958; Tasaki and Spyropoulos, 1959; Salt et al., 1987; Wangemann, 1995). This cell syncytium contains an armamentarium of ion channels, pumps, transporters, tight and gap junction mounting a polarized K^+^ cycling to create electricity on which hearing depends (Spicer and Schulte, 1991; Wangemann et al., 1995; Marcus et al., 2002; Hibino et al., 2010; Adachi et al., 2013; Lang et al., 2013; Yoshida et al., 2016; Liu et al., 2017). How these cellular networks orchestrate these delicate functions and what roles individual cells play under different conditions and diseases needs further elucidation.

Novel techniques have been applied to study gene regulation in complex networks (Korrapati et al., 2019; Taukulis et al., 2021). Single-cell RNA sequencing can map the transcriptome of individual inner ear cells in mammalian models (Petitpré et al., 2018; Shrestha et al., 2018; Chen et al., 2019). It exposes a genetic diversity reflecting cellular specializations and interplay behind the generation of EP and auditory reception (Liu H. et al., 2014; Korrapati et al., 2019; Bazard et al., 2021; Taukulis et al., 2021). Disruption of genes encoding several ion-regulating proteins and channels are known to cause profound sensorineural hearing loss (SNHL) and corresponding deafness-associated genes have already been mapped and targeted. This may lead to new ways to restore dysregulations of cochlear ion and fluid homeostasis (Korrapati et al., 2019; Gu et al., 2021) including gene therapy (Kelsell et al., 1997; Tranebjærg et al., 1999; Forge et al., 2003; Chan and Chang, 2014; Mei et al., 2017). Recently, RNA sequencing has been used to molecularly classify mouse spiral ganglion nerve diversity (Petitpré et al., 2018; Shrestha et al., 2018). Such information may lead to an improved understanding of auditory nerve function and improve electric nerve stimulation with cochlear prostheses used to treat patients with hearing loss.

Studies have shown that morphological and molecular divergences also exist among species (Liu et al., 2009; Liu W. et al., 2021). Thus far, broad investigations of gene distribution and expression profiles in the human cochlea have not been presented. Human studies are necessary but demanding, since well-preserved tissue is difficult to obtain (Liu et al., 2016). Swift fixation and mild decalcification are critical for cell preservation.

Here, we used RNAscope^®^ ISH of unique archival paraformaldehyde-fixed sections of the human cochleae to analyze the Na/K-ATPase (NKA) gene transcripts *ATP1A1*, *ATP1A3*, and *ATP1B1*, encoding the Na/K-ATPase α1, α3, and β1 isoforms. It is a highly sensitive technique for detecting single RNA transcripts. Super-resolution structured illumination microscopy (SR-SIM) was used to visualize single gene transcripts that appeared as bright foci, while unspecific labeling produced diffuse signals. NKA is a key ion transporter which maintains the EP and has been investigated in different species by several authors with somewhat different results (Kuijpers and Bonting, 1969; Mees, 1983; Schulte and Adams, 1989; Ichimiya et al., 1994; Schulte and Steel, 1994; Keithley et al., 1995; Peters et al., 2001; Weber et al., 2001; McLean et al., 2009; Liu et al., 2019; Stephenson et al., 2021). Studies of NKA expression were performed previously in the human cochlea (Weber et al., 2001; Liu et al., 2019; Liu W. et al., 2021; Stephenson et al., 2021). NKA is extensively expressed in human spiral ganglion somata, supporting its role in the relay and repolarization after spike activation (Pivovarov et al., 2019). NKA generates trans-membrane electrochemical gradients by the active transport of Na^+^ and K^+^, regulated by phosphorylation/de-phosphorylation of the α-subunit (Mohan et al., 2019). The enzyme consists of three subunits α, β, and FXYD with four catalytic α isoforms as well as three β and seven FXYD subunits (Clausen et al., 2017). The α1 subunit is expressed in virtually all cells (Watts et al., 1991). The β form is believed to be involved in the folding and transport of the synthesized catalytic α subunits (Geering, 1991). Combinations of NKA subunits influence a cell’s ability to bind and transport Na^+^ and K^+^, including in the cochlea (Therien and Blostein, 1999; Crambert et al., 2000; Peters et al., 2001; Blanco, 2005) as well as in the endolymphatic sac (Nordström et al., 2019). Preliminary RNAscope^®^ results were presented using SR-SIM to localize the *ATP1A1* and *ATPA1B1* gene transcripts, encoding NKAα1 and β1 isoforms (Liu W. et al., 2021). Here, we extended these analyses to include *ATP1A3*.

## Materials and Methods

### RNAscope^®^ Protocol

A cochlea isolated from a 67 years old female patient with 85% speech discrimination was used to perform RNAscope ISH. Specimens were obtained at surgery for removal of large, life-threatening petroclival meningioma where the cochlea had to be sacrificed during trans-cochlear surgery. Re-routing of the facial nerve was performed. Fixed-frozen human tissue sections underwent pretreatment with H_2_O_2_ (10 min, RT) and protease III (30 min, 40°C). After protease III incubation, the sections were subjected to an RNAscope^®^ hybridization assay. The probes were designed and produced by Bio-Techne (Minneapolis, MN, United States) depending on the targets’ gene ID (Table 1). To start the hybridization, the RNA probe(s) (in our study, a fluid mixture of probes named the C1, C2, and C3 channels) was added to the slide with sections. Incubation was performed in a HybEZ™ Oven (Bio-Techne) for 2 h at 40°C. After hybridization incubation, the slides were washed using 1x RNA-scope^®^ Wash Buffer. Then, the sections were incubated with RNA-scope^®^ Multiplex FL v2 Amp 1, 2, and 3 (for 30, 30, and 15 min, respectively) sequentially at 40°C to amplify the signal. For signal development, RNAscope^®^ Multiplex FL v2 HRP-C1, HRP-C2, and HRP-C3 were added to the sections sequentially (incubation time 15 min) in our RNAscope^®^ Multiplex study. For revealing signals, TSA-diluted Opal™ 520, 570, and 690 fluorophores were added to sections after HRP-C1, C2, and C3, incubating the sections for 30 min each at 40°C. The Na/K-*ATP1B1*, Na/K-*ATP1A3* and Cx probes were assigned with different Opal™ fluorophores in our three channel experiment (Ref: 541391 RNAscope probe Hs-GJB6, Ref: 539891 RNAscope probe Hs-ATPase alpha 1, Ref: 568261 RNAscope probe Hs-ATPase beta 1, Ref: 432501 RNAscope probe Hs-ATPase alpha 3) (Table 1). After each fluorophore incubation and rinse with 1x RNAscope^®^ Wash Buffer, RNAscope^®^ Multiplex FL v2 HRP blocker was added and incubated in an oven for 15 min at 40°C. Finally, the sections were counterstained with 4′,6-diamidino-2-phenylindole (DAPI) and the slides cover-slipped with ProLong^®^ Glass Antifade Mountant (Thermo Fisher Scientific, Waltham, MA, United States). RNAscope^®^ ISH produces puncta of signals that represent a single mRNA transcript (Grabinski et al., 2015). The RNAscope^®^ Multiplex Fluorescent v2 assay provides high sensitivity and allows single-molecule detection of up to four RNA targets simultaneously (Wang et al., 2015).

**TABLE 1:** The mRNA probes used in the present investigation.

Gene	Species	Gene ID	Chromosome location	Cat#	Company
GJB6	h	10804	13q12.11	541391	Bio-Techne
ATP1B1	h	481	1q24.2	568261	Bio-Techne
ATP1A3	h	478	19q13.2	432501	Bio-Techne
ATP1A1	h	476	1p13.1	539891	Bio-Techne

### Ethics Statements

Human cochlear tissue was obtained, as demonstrated in prior studies (Tylstedt et al., 1997). The study was approved by the Local Ethics Committee (Etikprövningsnämnden Uppsala no. 99398, 22/9 1999, cont., 2003, no. C254/4; no. C45/7 2007, Dnr. 2013/190), and patient consent was obtained. The study adhered to the rules of the Declaration of Helsinki. Specimens were obtained from patients suffering from life-threatening petroclival meningioma compressing the brain stem and undergoing transcochlear surgery. The operations were performed at Uppsala University Hospital by a team of neurosurgeons and otoneurosurgeons.

### Transmission Electron Microscopy and Immunohistochemistry

For TEM, four archival specimens were re-analyzed that were previously processed at the ear laboratory of the Ear, Nose and Throat Department at Uppsala University Hospital. Techniques used for processing and thin-sectioning of these specimens were described in a previous publications (Tylstedt et al., 1997). Specimens were from individuals without hearing impairment and were obtained at surgery for removal of large, life-threatening petroclival meningioma where the cochlea had to be sacrificed during trans-cochlear surgery. Patient data including gender and pure tone thresholds were described (Tylstedt et al., 1997). For immunohistochemistry we re-analyzed cochlear specimens and performed additional sectioning, antibody labeling and staining as described in publication (Liu W. et al., 2021). Fixation, decalcification, embedment and cryosectioning were described in material and methods together with patient data including gender, pure tone thresholds and speech discrimination. This included antibodies against NKA α1, α2, β1, Na-K-Cl cotransporter (NKCC), occludin, lamininβ2, Cx30, and Cx26. Na/K-ATPase subtypes were identified using high-resolution and multi-channel SR-SIM at Uppsala University SciLifeLab facilities at BioVis^1^, (Liu et al., 2019). The results were compared and presented in parallel with results from RNAscope analyses.

### Imaging and Photography

RNAscope^®^ sections were first investigated with an inverted fluorescence microscope (Nikon TE2000) equipped with a spot digital camera with three filters (for emission spectra maxima at 358, 461, and 555 nm). The image processing software was NIS Element BR-3.2 (Nikon). Laser confocal microscopy was performed using the same microscope equipped with a three-channel laser emission system. The software used was Nikon EZ-C1 (ver. 3.80). We used an Elyra S.1 SIM system with a 63x/1.4 oil Plan-Apochromat objective (Zeiss, Germany) and an sCMOS camera (pco. edge) with ZEN 2012 software. Multichannel SR-SIM imaging was conducted with a laser and filter setup: 405 nm laser of excitation coupled with BP 420–480 + LP 750 filter, 488 nm laser of excitation with BP 495–550 + LP750 filter, 561 nm laser of excitation with BP 570–620 + LP 750 filter, and 647 nm laser of excitation with LP 655 filter. SR-SIM images were processed using ZEN software. The 3D reconstruction was performed with Imaris 8.2 (Bitplane, Zurich, Switzerland). The resolution of the SIM system SR-SIM had a lateral precision of 80 nm (Gustafsson et al., 2008). The resolution was measured with subresolution fluorescent beads (40 nm; Zeiss) in the green channel (BP 495–550 + LP750). An average PSF value was obtained from multiple beads using the built-in experimental PSF algorithm of the ZEN software.

## Results

### Cochlear Duct Epithelium and *ATP1A1* and *ATP1B1*

**FIGURE 1:**
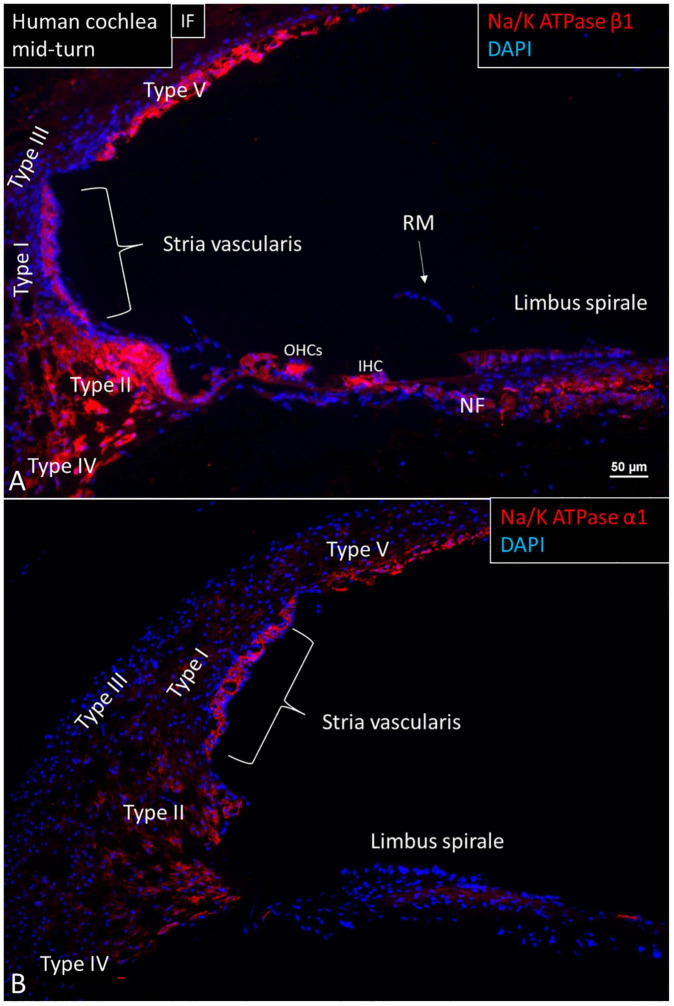
Immunofluorescence (IF, **A**) and confocal microscopy **(B)** of cross-sectioned human cochlea shows expression of NKAβ1 and NKAα1. The marginal cells, type II, IV, and V fibrocytes, outer sulcus, Hensen cells, interdental cells, spiral limbus fibrocytes, and auditory neurons express NKAβ1 but not type I and III fibrocytes, RM and the tympanic covering layer. The NKAα1 subunit is strongly expressed in the SV marginal cells but less in type IV and V fibrocytes. OHCs, outer hair cells; IHC, inner hair cell.

**FIGURE 2:**
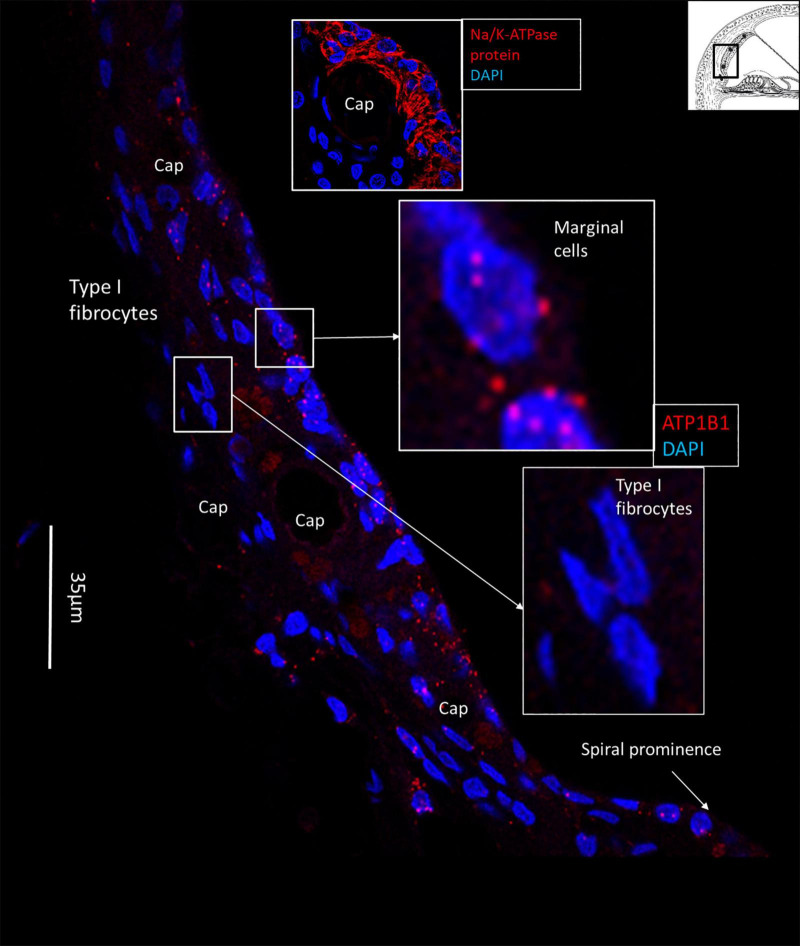
SR-SIM of the human lateral cochlear wall. *ATP1B1* gene transcripts are mainly expressed in marginal cells. Type I fibrocytes and blood vessels lack expression of *ATP1B1*. Some cells located near the spiral prominence contain *ATP1B1* gene transcripts. Right inset shows expression of *ATP1B1* in marginal cells. Left inset shows basal cells at higher magnification with no ATP1B1 gene transcripts visible.

**FIGURE 3:**
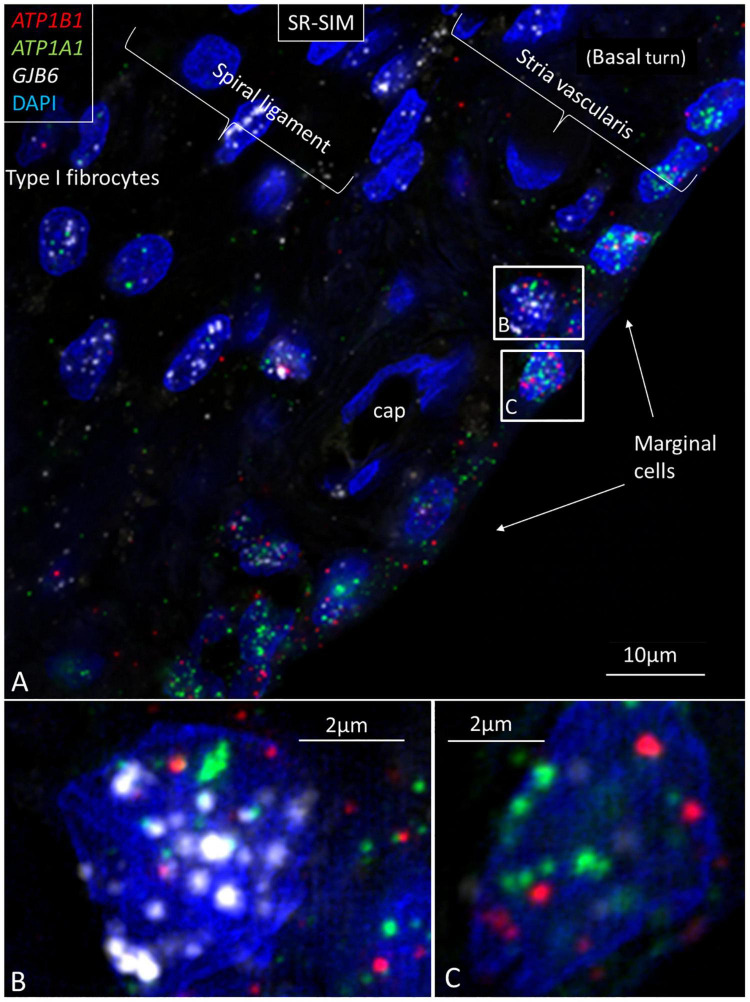
Multiplex RNAscope^®^ shows *ATP1A1*, *ATP1B1*, and *GJB6* gene transcripts in the lateral wall of the human cochlea. **(A)** Marginal cells express *ATP1A1* and *ATP1B1* genes, while basal and intermediate cells mainly express the *GJB6* encoding the protein Cx30. A few cells also express single NKA gene puncta. A capillary in the SV contains no gene transcripts. **(B)** Framed cell in **(A)** is shown at higher magnification. The cell contains all three gene transcripts in the cell nucleus. **(C)** Framed marginal cell in **(A)** is shown at higher magnification. The nucleus contains *ATP1A1* and *ATP1B1* genes, but no GJB6. DAPI, 4′,6-diamidino-2-phenylindole, a cell nucleus marker.

**FIGURE 4:**
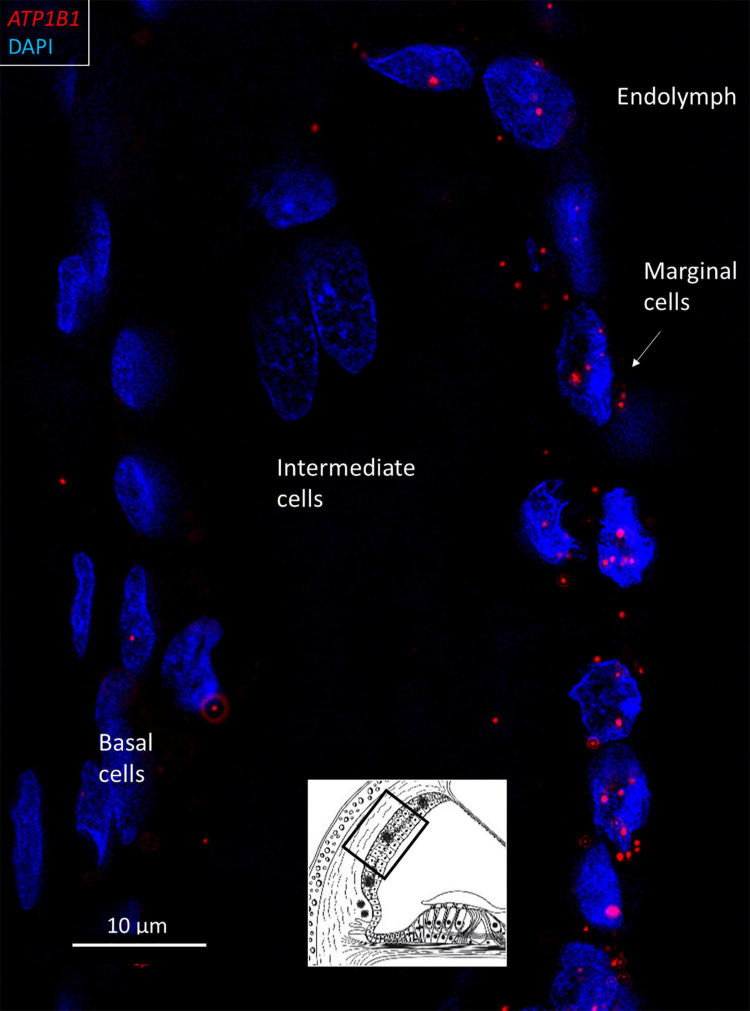
SR-SIM of *ATP1B1* gene transcripts (red spots) expressed in the marginal cells of the SV in the basal turn of the human cochlea (inset). Gene expression is restricted to the cells contacting endolymph, with only a few transcripts present in the intermediate and basal cells.

The expression of NKAβ1 and NKAα1 proteins in the human cochlea is shown in Figure 1. *ATP1A1* and *ATP1B1* mRNA transcripts were detected as red puncta in the cochlear duct epithelial cells, except in Reissner’s membrane (RM). The number of puncta varied in different domains. In the SV, only the marginal cells contained *ATP1A1* and *ATP1B1* mRNA transcripts consistent with protein expression at immunohistochemistry (Figures 2–4). No transcripts were detected in the intermediate and basal cells. In the marginal cells, *ATP1A1* and *ATP1B1* were located both in the cytoplasm and cell nuclei (Figures 3, 4). The transcripts were often found at the rim of the cell nucleus, indicating its transport through the nuclear pores out into the cytoplasm. *GJB6* mRNA transcripts were found in the basal, intermediate cells of the SV and type I fibrocytes, consistent with their protein location (Figure 4). They were not observed in the marginal cells and served as controls. No expression of *ATP1A1* and *ATP1B1* was found in the SV blood vessels.

**FIGURE 5:**
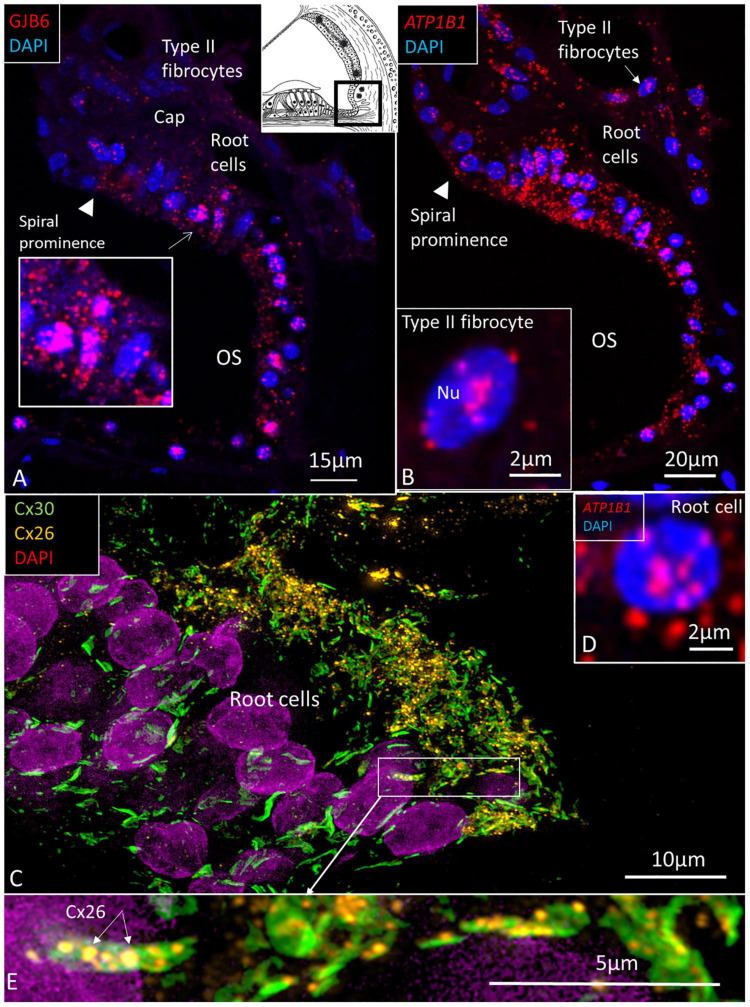
**(A)** The human outer sulcus epithelium demonstrates many red puncta representing GJB6 transcripts. These are also seen in type II fibrocytes. Inset shows root cells at higher magnification. **(B)** *ATP1B1* gene transcripts are also expressed in the outer sulcus epithelium and in type II fibrocytes. Inset shows a type II fibrocyte at higher magnification with mRNA transcripts located in the nucleus (Nu) and at nuclear pore regions. **(C)** SR-SIM shows expression of Cx26 and Cx30 in the root cells. Inset **(D)** shows a root cell expressing *ATP1B1*. Framed area is magnified in **(E)**, showing several large Cx30 GJ plaques in the root cells and associated Cx26 plaques (arrows). OS, outer sulcus; Cap, capillary.

**FIGURE 6:**
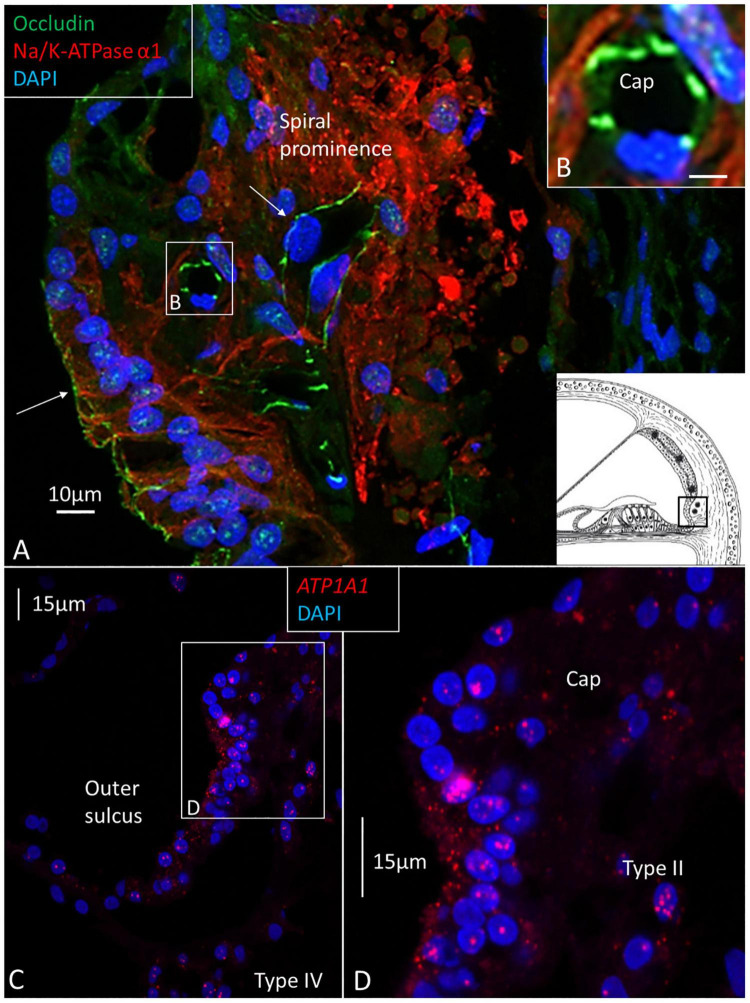
**(A)** Confocal immunohistochemistry of human spiral prominence. The tight junction protein occludin is expressed in epithelial cells and capillary endothelium (Cap) (arrows). The basolateral cell membrane of the epithelial and type II cells express NKAα1. Framed area is magnified in inset **(B)** and shows expression of occludin. NKAα1 and occludin are separately expressed. **(C)** RNAscope^®^ and SR-SIM of outer sulcus and spiral prominence show expression of *ATP1A1* in the epithelium and type II fibrocytes. A few gene puncta are also seen in the type IV fibrocytes. Framed area is magnified in **(D)** and shows *ATP1A1* richly expressed in the outer sulcus epithelium, root cells, and type II fibrocytes. There are no *ATP1A1* transcripts in the blood capillary.

The largest number of *ATP1B1* and *ATP1A1* transcripts were present in the outer sulcus epithelium, root cells, and spiral prominence epithelium (Figures 5, 6). The transcripts were only expressed in the lower part of the spiral prominence. The upper part facing the SV differed in morphology, being flatter and more electron dense as shown by TEM (Liu et al., 2016). The outer sulcus cells and root cells contained a large number of *GJB6* mRNA transcripts consistent with prominent gap junction (GJ) plaques expressing Cx30 (Figure 5E; Liu et al., 2017). Multiplex RNAscope^®^ showed that these cells contained *GJB6* transcripts with almost identical distribution of *ATP1B1* and *GJB6* (Figures 5A,B). The RNAscope^®^ of the outer sulcus and spiral prominence showed that *ATP1A1* was richly expressed in the outer sulcus epithelium, root cells, and type II fibrocytes (Figure 6). A few gene transcripts were detected in type IV fibrocytes. There were no *ATP1A1* transcript genes in the blood capillary.

**FIGURE 7:**
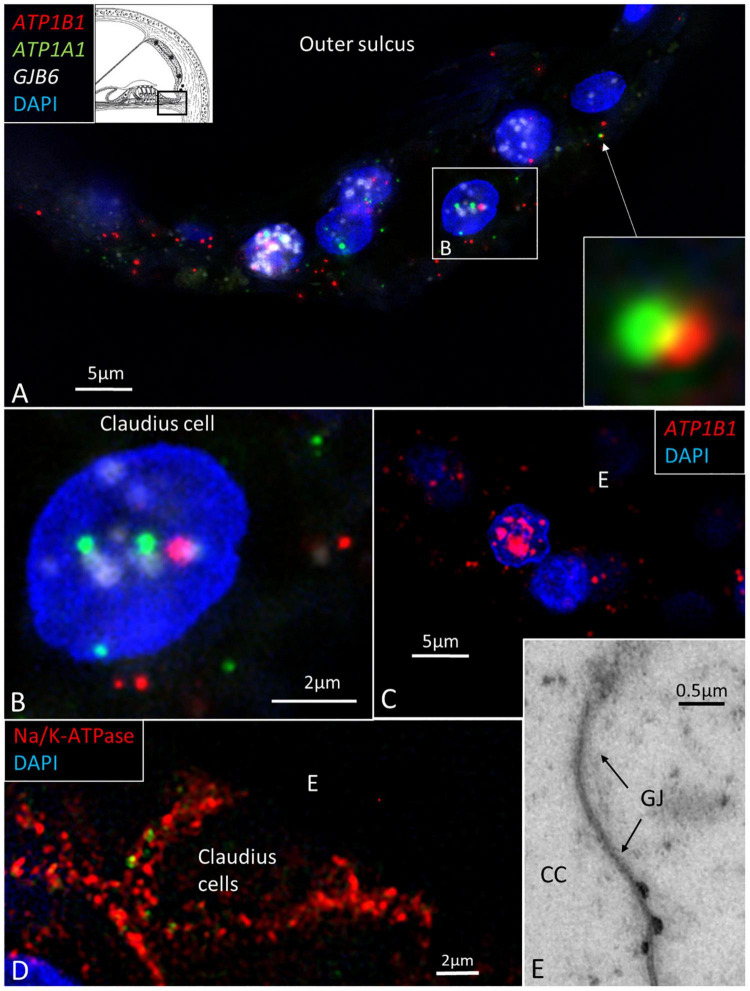
**(A)** SR-SIM of Claudius cells in a human cochlea. *ATP1B1*, *ATP1A1*, and *GJB6* gene transcripts are expressed in cell nuclei and cytoplasm. Two gene puncta appear close to each other. Framed area is magnified in **(B)**. **(B)** Cell nucleus contains all three genes. **(C)** Claudius cell nucleus contains a large number of *ATP1B1* gene puncta. **(D)** Claudius cell expresses Na/K-ATPaseβ1 in the basolateral cell membrane. **(E)** TEM shows a GJ between two Claudius cells.

In the spiral prominence, many *ATP1B1* and *ATP1A1* transcripts were located in the Claudius and Boettcher cells (Figures 7A–E). Occasionally, some *ATP1A1* and *ATP1B1* gene transcripts were located closely together (Figure 7A, inset).

### Organ of Corti and Spiral Limbus

**FIGURE 8:**
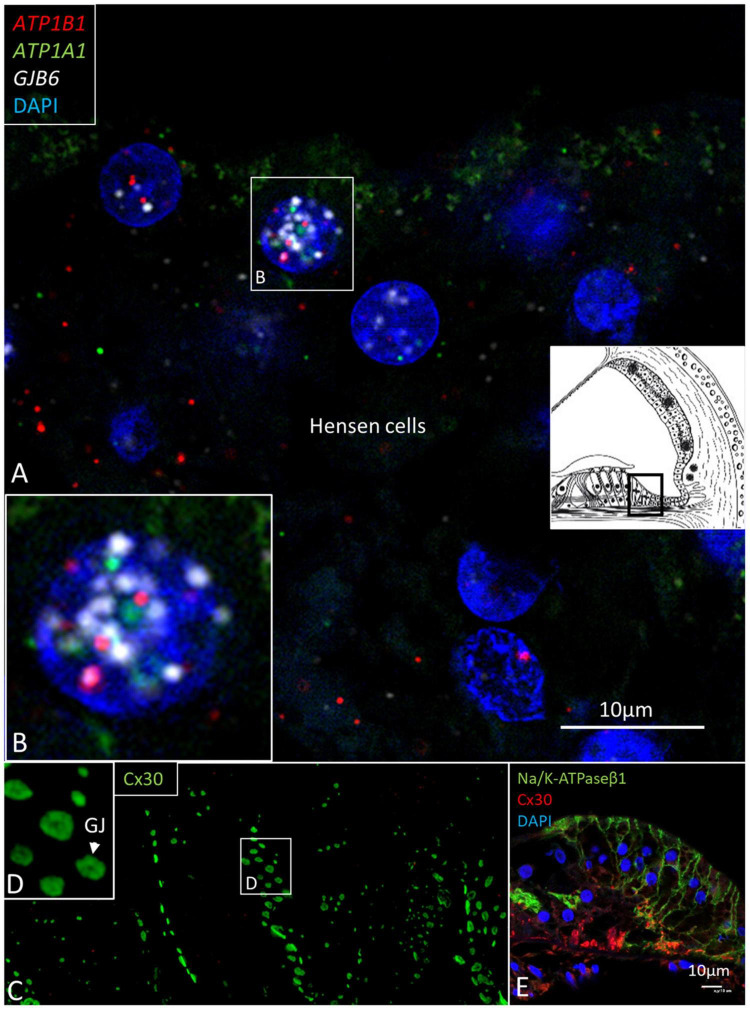
**(A)** Multiplex RNAscope^®^ ISH and SR-SIM of the Hensen cell region. Cell nuclei contain many *ATP1A1*, *ATP1B1*, and *GJB6* gene transcripts. Framed area is magnified in **(B)**. **(B)** The 1cell nucleus contains all three transcripts, suggesting active transcription of NKA and Cx30. **(C)** SR-SIM of Cx30-positive GJ plaques (green) are seen between Hensen cells. **(D)** Framed area in **(C)** is magnified. **(E)** Confocal microscopy showed expression of NKAβ1 in the lateral cell membranes of Hensen cells. Cx30 was also expressed.

**FIGURE 9:**
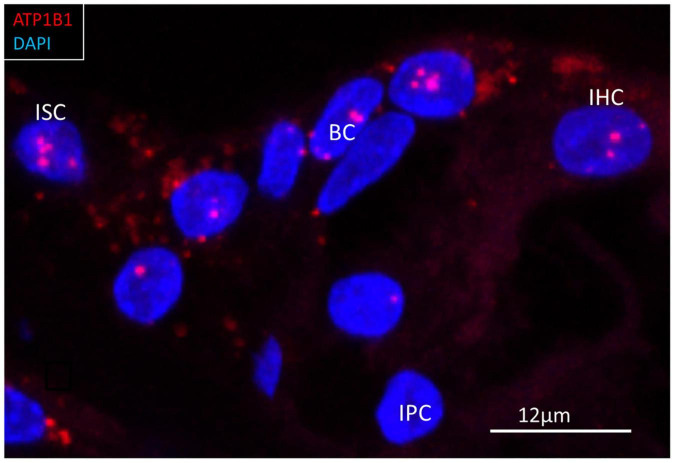
RNAscope^®^ of *ATP1B1* transcripts in the region of a human inner hair cell (IHC). Gene transcripts are seen in the supporting cells around the IHC. One IHC contained a few transcripts. ISC, inner sulcus cell; IPC, inner pillar cell; BC, border cell.

*ATP1B1* and *ATP1A1* gene transcripts were detected in border, phalangeal, pillars, Deiters, and Hensen cells in the cell cytoplasm and nuclei (Figures 8, 9). The findings are consistent with NKA β1 expressed in the basolateral cell membranes of the supporting cells (Liu et al., 2019). Most mRNA transcripts were located in the cell nuclei, indicating active gene transcription (Figures 8A,B). The large number of *ATP1B1* and *ATP1A1* transcripts in Hensen cells is consistent with the rich protein expression (Figures 8D,E). The cell nuclei of the inner hair cells (IHCs) contained sporadic *ATP1B1* transcripts (Figure 9). No *ATP1B1* or *ATP1A1* gene transcripts were found in myelinated axons beneath the habenula perforata or in nerve fibers in the organ of Corti, such as the afferent terminals of the inner and outer hair cells, inner and outer spiral bundles, tunnel spiral, or tunnel crossing fibers. Cells located beneath the basilar membrane occasionally contained conglomerates of *ATP1B1* gene transcripts in the nuclei. The inner sulcus and interdental cells contained a few *ATP1A1* and *ATP1B1* gene transcripts.

### Spiral Ligament

No *ATP1B1 or ATP1A1* mRNA transcripts were found in type I and III fibrocytes. Type V fibrocytes contained *ATP1A1* and *ATP1B1* transcripts, consistent with protein localization. Type IV fibrocytes contained few *ATP1A1* and *ATP1B1* transcripts. Also, type II fibrocytes, particularly those facing the root cells, contained a large number of *ATP1B1 and ATP1A1* transcripts (Figures 5B, 6C,D). The transcripts appeared mostly in the cell nuclei and in nuclear pore regions. A large number of *GJB6* transcripts were also located in the type II fibrocyte cell nuclei, but there were also plenty of these gene transcripts located in the cytoplasm (Figure 5A). They closely matched the location of the *ATP1A1* and *ATP1B1* gene transcripts.

### Spiral Ganglion

**FIGURE 10:**
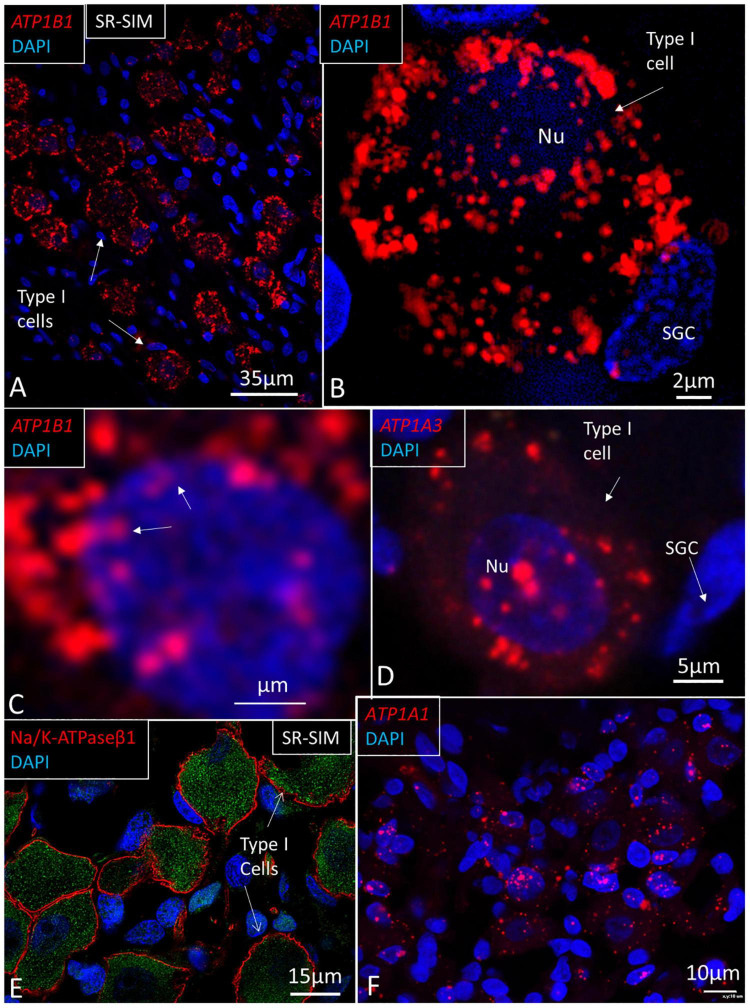
SR-SIM of human spiral ganglion. **(A)** Type I spiral ganglion cells display a large number of red *ATP1B1* gene puncta. **(B)** Higher magnification of a type I cell shows gene transcripts in the cell nucleus (Nu) and cytoplasm. **(C)** *ATP1B1* gene transcripts are seen at the periphery of the cell nucleus, assumingly at the nuclear pores (arrows). **(D)** *ATP1A3* transcripts in a type I spiral ganglion cell. **(E)** SR-SIM of type I ganglion cells shows NKAβ1 expression along the plasma lemma. **(F)** *ATP1A1* gene expression in Rosenthal’s canal. Both ganglion cells and adjacent cells show the expression of *ATP1A1*. SGC, satellite glia cell.

**FIGURE 11:**
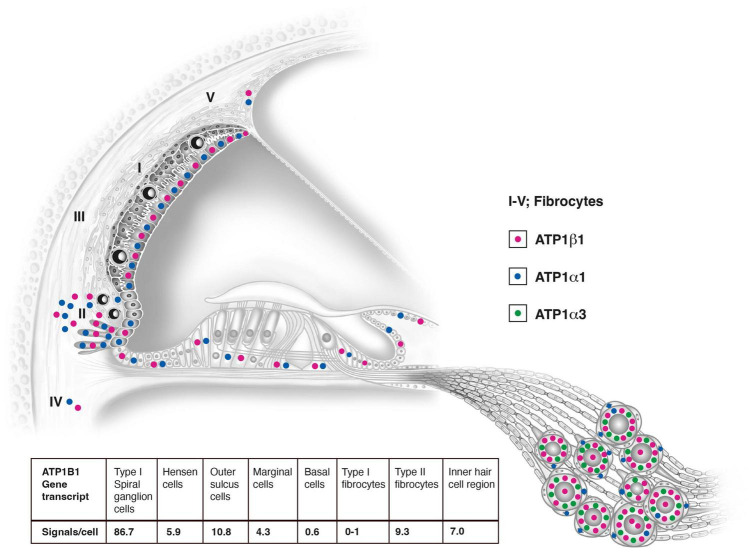
Relative distribution of *ATP1A1*, *ATP1A3*, and *ATP1B1* gene transcripts in the human cochlea based on RNAscope^®^ ISH. Gene transcription may suggest that the lateral pathway of K^+^ cycling from the organ of Corti is dominant. Type I spiral ganglion cells contain the largest number of mRNA gene puncta. They overcame signal counts in marginal cells 20 times and type II fibrocytes nine times. The number of counts in the ganglion cells was probably higher since many puncta were concentrated in clusters due to their large number near the plasmalemma. Results of quantitative analysis of ATP1B1 signal counts per cell in different cellular domains of the human cochlea are shown and in Table 2.

The largest number of *ATP1B1* gene transcripts was found in the type I spiral ganglion cells; *ATP1B1* signal counts exceeded those in marginal cells 20 times and type II fibrocytes nine times (Table 2 and Figures 10A–C, 11). They also contained many *ATP1A3* transcripts (Figure 10D). No other cells in the Rosenthal’s canal or in the cochlea displayed *ATP1A3* gene transcripts. *ATP1B1* gene transcripts were observed in cell nuclei and cytoplasm, often assembled in larger conglomerates near the plasma membrane where NKAβ1 protein was highly expressed (Figure 10E). If type II spiral ganglion cells expressed *ATP1B1* and *ATP1A3* gene transcripts could not be assessed with certainty since a specific marker for these cells was not used. Satellite cells lacked *ATP1B1* but contained *ATP1A1* (Figures 10F, 11). Type I cells also contained *ATP1A1* transcripts, as well as some surrounding mesenchymal cells (Figure 10E). Axons lacked *ATP1A1*, *ATP1B1*, and *ATP1B3* gene transcripts, while NKAβ1 protein was richly expressed along the axonal plasma membrane in both myelinated and unmyelinated fibers. The *GJB6* labeling yielded uncertain results. A Supplementary Video 1 shows *ATP1B1* mRNA transcripts in human type I spiral ganglion cells from seven slices (movie). The relative expression of gene transcripts is shown in Table 3.

**TABLE 2:** Results of quantitative analysis of ATP1B1 signal counts per cell in different cellular domains of the human cochlea.

ATP1B1 gene transcript	Type I spiral ganglion cells	Hensen cells	Outer sulcus	Marginal cells	Basal cells	Type I fibrocytes	Type II fibrocytes	Inner hair cell region
Signal counts/cell	86.7	5.9	10.8	4.3	0.6	2.3	9.3	7.0

**TABLE 3:** Relative expression of gene transcripts.

	ATP1A1	ATP1B1	ATP1A3	GJB6
OSC	+	+++	−	++
HC	++	+++	−	++
RC	++	+++	−	++
Type I	(−)	(−)	−	++
Type II	++	+++	−	++
Type III	−	−	−	−
Type IV	(−)	+	−	+
Type V	(−)	+	−	+
MC	++	++	−	−
IC	(−)	(−)	−	+
BC	(−)	(−)	−	++
SG (I)	+	+++	++	?
SGC	+	−	−	−

*OSCs, outer sulcus cells; HC, Hensen cells; RC, root cells; MC, marginal cells; IC, intermediate cells; BC, basal cells; Types I–V, fibrocytes; SG (I), spiral ganglion cells type I; SGC, satellite glia cell; +, positive expression; −, no expression; ?, occasional puncta.*

## Discussion

RNAscope^®^ ISH using target-specific probes localized and quantified single NKA mRNA species in formaldehyde-fixed sections of surgically obtained human cochleae (Wang et al., 2012; Bingham et al., 2017; Duncan et al., 2019). Rapid fixation and mild decalcification maintained *ATP1A1*, *ATP1B1*, *ATP1A3*, and *GJB6* mRNA transcripts and cell integrity. Control tagging and protein congruity validated the specificity. A limitation was the small amount of human tissue restricting more systematic gene localization along the cochlear spiral.

### Lateral Cochlear Wall and NKA Gene Transcription

The results showed that the number of NKA gene transcripts varied highly in the cellular domains of the lateral cochlear wall. This confirmed that *ATP1A1* and *ATP1B1* are not expressed in the basal and intermediate cells in the human SV, but only in the marginal cells responsible for the secretion of K^+^ into endolymph. The spiral ligament contains mesenchymal cells (type I–V fibrocytes) with high ion transport activity believed to be important for K^+^ cycling (Spicer and Schulte, 1991, 1996). No NKA gene transcripts were seen in type I fibrocytes connected to the basal cells of the SV through multiple GJs. A surprisingly large number of *ATP1A1* and *ATP1B1* gene transcripts were detected in the outer sulcus epithelium, root cells, and type II fibrocytes, the largest in the cochlear duct. There were more than twice as many as in the marginal cells of the SV (ratio 10.8/4.3) (Table 2). Moreover, the cells contained a large number of *GJB6* transcripts consistent with the high expression of Cx30 protein and distribution of GJ channels in humans (Liu et al., 2016). This suggests that these cells may play a major role for K^+^ cycling. A few NKA gene transcripts were present in type IV and V fibrocytes.

*ATP1A1*, *ATP1B1*, and *GJB6* gene transcripts were not detected in SV vessels. Prior studies have shown that the α1 and β1 subunits are not associated with endothelial tight junctions and occludin (Liu et al., 2017). The NKA α1 subunit was previously identified in isolated mouse SV to interact with occludin and could be essential for the integrity of the blood-labyrinth barrier (Yang et al., 2011). This barrier may be challenged by noise and other factors, leading to increased vascular leakage and hearing loss. Yet, we found that α1 and occludin were expressed in the endothelium of the spiral prominence vessels surrounded by type II fibrocytes, which also expressed α1. This suggests that the ion and fluid permeability of these vessels may be higher compared with the SV vessels. Type III fibrocytes located near the outer bony wall contained no *ATP1A1* or *ATP1B1* gene transcripts, suggesting that these cells are not involved in K^+^ transport to a major extent. In the gerbil cochlea, these cells were found to express creatine kinase and carbonic anhydrase (Spicer and Schulte, 1991).

### K^+^ Cycling

It is generally believed that there is both a medial and lateral transcellular pathway for the uptake and transport of K^+^ from the organ of Corti during auditory transduction. The lateral route consists of a transcellular passage from the outer hair cells to the Deiters, Hensen, Claudius, outer sulcus, and root cells back to the subepithelial space upheld by NKA activity in the type II fibrocytes. From there, K^+^ may flow through GJs to type I fibrocytes and basal and intermediate cells to the intrastrial compartment (Weber et al., 2001). A similar route was suggested medially from the inner hair cells via the inner sulcus, spiral limbus, and interdental cells. Our results support the lateral cycling of ions via the outer sulcus. Hensen and Deiters cells contained a large number of *ATP1A1*, *ATP1B1*, and *GJB6* transcripts, with several puncta located in the cell nuclei suggesting active transcription. Type II fibrocytes strongly express NKCC1 that takes up and recycles K^+^ ions essential for the high intracellular concentration in the intermediate cells in the SV and generation of the EP (Wangemann et al., 1995; Crouch et al., 1997; Miuta et al., 1997; Weber et al., 2001; Liu et al., 2017). NKA may play a lesser role for recycling of K^+^ from type I fibrocytes back to the SV. Instead, these cells may shunt ions across the large number of Cx30-positive GJ channels, which is also reflected by the large number of *GJB6* gene transcripts. Weber et al. (2001) only detected the β2 subunit in type II, IV, and V fibrocytes and limbal fibrocytes but not in type I cells (Weber et al., 2001). In a more recent study, α1 was localized in type I, II, and IV fibrocytes in the human cochlea (Stephenson et al., 2021). In the present study the expressed gene transcripts matched the protein distribution, validating the *in situ* hybridization, and *GJB6* localization functioned as an additional positive control. A more systematic analysis of the *GJB2* and *GJB6* gene transcripts encoding proteins Cx26 and Cx30 will be performed in a separate study.

Few *ATP1B1* and *ATP1A1* transcripts were detected in the inner sulcus epithelium, limbus fibrocytes, and interdental cells. SR-SIM previously demonstrated β1 combined with α1 (Liu et al., 2019) and the non-sensory cells contained *GJB6* and a large number of Cx30-positive GJ channels. Yet, a comparable large number of NKA gene transcripts could not be observed medially along the border cells, inner sulcus, and spiral limbus. Moreover, only a limited number of gene transcripts were verified in the scala vestibuli and scala tympani wall (type IV and V fibrocytes) which are believed to play a role in endolymph and perilymph K^+^ recirculation. The findings suggest that the lateral pathway of transcellular K^+^ flux is more significant and also recycles K^+^ from the medial hair cell region.

### Reissner’s Membrane

The RM sustains steep ion gradients between the endolymph and perilymph. The epithelium contains tight junction occludin (Liu et al., 2017). The epithelium was shown to specifically absorb Na^+^ from endolymph contributing to the exceptional low concentration of Na^+^, a prerequisite for normal cochlear function (Lee and Marcus, 2003). No *ATP1B1* or *ATP1A1* gene transcripts were detected in the RM, which agrees with the lack of α1 and β1 protein expression (Liu et al., 2019) even though alternate subunit isoforms could exist. Results from ultramicro enzyme assay techniques suggested that the NKA contribution in the RM is minor (Kuijpers and Bonting, 1969). This was also supported by its high electrical resistance (Naftalin and Harrison, 1958).

### Human Auditory Nerve and NKA Gene Transcription

The large number of *ATP1A3* and *ATP1B1* gene transcripts in type I spiral ganglion cell bodies suggest an extraordinary transcription rate and production of NKA α3 and β1 proteins in the human cochlear nerve (Figure 11). This is consistent with the major effects of a dominant, missense mutation in the *ATP1A3* gene on chromosome 19q13.2, encoding the neuron-specific α3 subunit (De Carvalho Aguiar et al., 2004; Demos et al., 2014; Tranebjærg et al., 2018). Notably, both transcripts aggregated near the plasmalemma where α3 and β1 subunits are heavily expressed. NKA activity depends on the catalytic α-subunit, while the β-subunit may act as a chaperone for proper translation, folding, and transport from the rough endoplasmic reticulum (rER) to the enclosure of the α-subunit in the cell membrane (Geering, 1991). Nerve perikarya hold extensive rER and free ribosomes, and mitochondria occupy nearly 25% of the cytosol, suggesting a high energy consumption. These are typical features in anoxia-sensitive cells with large ion fluxes related to excitation and conduction (Ames, 2000; Howarth et al., 2012). Auditory neurons have a rich energy demand due to their high spontaneous electric activity to fully, and with low thresholds, replicate the acoustic signals. Neuronal NKA activity depends on continuous energy supplied from intracellular ATP, and oxygen deprivation leads to inhibition of NKA, increased cytosolic Na^+^ concentration, and neuronal dysfunction (Haddad and Jiang, 1993).

While the human spiral ganglion neurons express β1 and α3 subunits, the surrounding unmyelinated satellite glia cells express the α1 isoform (Liu et al., 2019). This is consistent with the distribution of *ATP1A1* in satellite cells. *ATP1A1* transcripts were also found in type I ganglion cell bodies, explained by different regulatory pathways. Our findings indicated that *ATP1A3* and *ATP1A1* mRNA species are not derived from interconversion or posttranslational modification but are separate gene transcripts. Neurons may use the α1 NKA as a “housekeeping” transporter to maintain basal ionic gradients (Dobretsov and Stimers, 2005), whereas during high-frequency activity, α3 may be activated to reestablish the membrane potential after repetitive spike generation, potentially increasing cytosol Na^+^ concentration. The ionic gradients of the fast-spiking neurons need to be restored swiftly after prolonged bursts of action potentials to optimize signal precision, partly by modulating calcium buffering during hyperpolarization (Dobretsov and Stimers, 2005; Kim et al., 2007).

### Axonal Transport

No *ATP1B1* or *ATP1A3* gene transcripts were found in axons or neurons beneath the inner and outer hair cells, suggesting that proteins may be synthesized outside the organ of Corti, presumably in the spiral ganglion. Both β1 and α3 are richly expressed in neurons in the organ of Corti (Liu W. et al., 2021). The paucity of gene transcripts suggests that proteins are synthetized and transported from the cell soma to the axons and nerve terminals, whose function would depend on rapid axonal transport, partly through the microtubule system (Nakata et al., 1998; Ahmari et al., 2000; Dobretsov and Stimers, 2005). In the rat optic nerve, retinal ganglion cells synthesize and transport both catalytic NKA subunits with few signs of local synthesis. Yet, in humans, electron microscopy findings suggest that the peripheral neural processes hold the machineries for mRNA translation. Mitochondria and monoribosomes are located in the axonal initial segments along the internodal spaces and in nerve terminals, which was also verified among *in vitro* cultured growth cones (Tylstedt et al., 1997; Anderson et al., 2006; Liu W. et al., 2021). This suggests that there is a distal transport of transcriptome modules with mRNA for local translation in the axonal processes. This may serve different functions, such as axon guidance, navigation, synapse formation, regeneration, and even mitochondrial function (Kaplan et al., 2009). Proteins could also be modified during axonal transport, such as for peptide processing, proteolysis, and axonal glycosylation (Specht and Sweadner, 1984). In the rat, NKA α3 is abundantly expressed in type I afferent terminals and the medial efferent terminals at the outer hair cells (McLean et al., 2009). NKA α1 is also expressed in supporting cells at the inner hair cells that express the glutamate transporter GLAST, conceivably acting in concert for reuptake of transmitter substance to regulate afferent signaling. The results suggest that both α1 and α3 play roles in the regulation of the type I afferent synapses, the medial efferent synapses, and glutamate transport from the afferent inner hair cell synapse (McLean et al., 2009). Multiple levels of the mRNA α3 isoform were found in the spiral ganglion but at no other locations (Ryan and Watts, 1991) while ten Cate et al. (1994) found high levels of β1 and α3 and low levels of the α1 at the base of the inner hair cells in the rat cochlea and the cochlear nerve (ten Cate et al., 1994). In the gerbil, spiral ganglion neurons and their peripheral processes showed positive immunostaining of β1 and α3, but no α1 in the cochlear nerve endings (Mcguirt and Schulte, 1994). We found that the nerve fibers within the human organ of Corti express both α1 and α3 isoforms as well as β1, which are assumed to play a role in the fine regulation of nerve signaling in man (Liu et al., 2019).

### Spiral Ganglion Neuron Heterogeneity

Single-cell RNA sequencing was used to molecularly classify murine type I SGNs into three subtypes expressing combinations of Ca^2+^ binding proteins, ion channel regulators, guidance molecules, and transcription factors (Shrestha et al., 2018). The remarkable electrophysiological heterogeneity, further specified by neurons tonotopic location, may serve to increase the dynamic range and temporal signal acuity by representations of molecularly different SGNs (Mo and Davis, 1997; Taberner and Liberman, 2005; Liu Q. et al., 2014; Shrestha et al., 2018). Complex sounds could be deciphered by different subtypes and their relative numbers along the frequency axis. The *ATP1B1* gene transcript was evenly distributed along the cochlear spiral. We could not determine if the distribution of NKA gene transcripts varied along the cochlear spiral, partly due to the limited amount of tissue. Hopefully, further analyses will be performed to verify whether NKA expression varies along the spiral and if different molecular subtypes exist among the type I SGNs in humans. Such a study should also include different combinations of voltage-gated potassium channels (Kv1) and hyperpolarization-activated cyclic nucleotide-gated channel α-subunits 1–4 (HCN1 and HCN4). Moreover, the study should include sodium-activated potassium K_Na_1 that may play a protective role in neurons by reducing membrane electrical activity and prevent intracellular Ca^2+^ accumulation (Yuan et al., 2003). RNA sequencing and single molecule fluorescence *in situ* hybridization (smFISH) were used to localize transcripts encoding the K_Na_1 channels and their influence on NKAα3 expression in murine SGNs (Reijntjes et al., 2019). K_Na_1 was found to be essential for normal cochlear function and activity of the primary auditory neurons, but removal of these channels did not alter NKAα3 expression.

### Sodium-Potassium Pump – A Spike Integrator?

In the brain, NKA was proposed to act not only as a “housekeeping” enzyme but also as an information processing element by controlling intrinsic firing and coding in cerebellar Purkinje neurons (Forrest et al., 2012). Pump activity depends on Na^+^ level, which could act as a memory element to record firing history to be used to monitor spontaneous activity (Forrest, 2014). Evidence was obtained that NKA could memorize voltage history and act as a spike integrator, modulating timing, amplitude, bursting, and quiescence *in vitro* by hyperpolarizing the cell membrane. A corresponding spike generator/integrator at the level of the SGN cell soma or groups of cells could source information to increase the dynamic range and even decipher complex acoustic message information. This could equally explain the unique properties of the human auditory nerve to decipher complex auditory information from electric stimulation in cochlear implant recipients, even after bypassing the tuned auditory receptors and peripheral processes. Human SGN cell bodies are endowed with voltage-gated ion channels that may act as spike generators and vary along the cochlear spiral (Liu W. et al., 2021). Potassium channels may play an essential role for these variable characteristics, providing diverse firing patterns, including thresholds and accommodation (Davis and Crozier, 2015). Kv1 currents appear in unmyelinated regions, including the juxtaparanode (Chiu and Ritchie, 1980; Liu W. et al., 2021). Human SGN cell soma lack myelin and, at some places, even satellite cells, allowing direct physical interaction between clustered cells, especially in the apical region. Human cell bodies could act as Ranvier nodes, and the electric coupling could synchronize APs, similar to the firing in Purkinje neurons (Han et al., 2018). NKA α1 and β1 as well as HCN channels 1, 2, and 4 are richly expressed at adjoining cell membranes in the apical cochlea (Liu et al., 2019; Luque et al., 2021). HCN channels may generate “pacemaker currents” in heart muscle cells and could act to boost coupling and synchronize firing, similar to that described in the brain. A preprocessing of phase characteristics was suggested to establish low-frequency phase-locking combining place and rate coding essential for the reception of human speech (Luque et al., 2021). The RNAscope^®^ also localized *ATP1B3* transcripts in the cell nuclei in the large type I ganglion cells (Liu et al., 2020). β3-subunits could assist in “gluing” cells together (Geering, 1991) for clustering. Different isoforms were previously found to be co-expressed in the same cell in the human brain (Tokhtaeva et al., 2012).

### Sodium-Potassium Pump and Inner Ear Disease

Molecular studies, including immunocytochemistry and gene targeting, indicate that age-related hearing loss (ARHL) may be associated with a reduction of NKA activity in the SV and spiral ligament (Schulte and Schmiedt, 1992; Gratton and Schulte, 1995; Lippincott and Rarey, 1997; Diaz et al., 2007; Ding et al., 2018; Liu Y. et al., 2021; Stephenson et al., 2021). Both α1 and α2 subunit isoforms were analyzed as well as β1 and β2. A dysregulation of NKA activity was also suggested to be associated with the binding between the α1 and β1 heterodimer subunits (Ding et al., 2018). The spiral ligament cells may be dysregulated by inflammatory changes, and GJ changes may disrupt K^+^ recycling and cause impaired endolymph homeostasis and sensorineural hearing loss (SNHL; Schulte and Schmiedt, 1992; Kelsell et al., 1997; Ichimiya et al., 2000; Kitajiri et al., 2004; Furness, 2019; Bazard et al., 2021; Liu Y. et al., 2021; Stephenson et al., 2021). Loss of SGNs is also known to occur in ARHL and can be independent of sensory hair cell loss (Schuknecht and Gacek, 1993). It may have both a genetic and an environmental background (Perez et al., 2011). It has not been determined whether reduced NKA activity or gene dysregulation in the SGNs is associated with ARHL. The high spontaneous rate may require a swift production of protein, reflected in a high transcription rate widely surpassing the marginal cells. Reduced activity would result in an impaired reset of ionic concentration, decreased spike rate, and signal dysregulation. Human cell bodies are remarkably resistant and often, unlike most mammals, preserved even after loss of hair and supporting cells and even peripheral axons in humans (Linthicum and Fayad, 2009). This is important for patients with cochlear implants whose electric stimulation requires effectiveness even after long-term use. If the high *ATP1A3* and *ATP1B1* transcription rate plays a role in functional preservation remains to be elucidated. Nonetheless, a better understanding of the molecular background of SGN and lateral wall dysfunction could lead to new innovative therapies to restore hearing in the large population suffering from ARHL. Such strategies could include both gene- and cell-mediated therapy.

## Data Availability Statement

The original contributions presented in the study are included in the article/Supplementary Material, further inquiries can be directed to the corresponding author.

## Ethics Statement

Human cochlear tissue was obtained, as demonstrated in prior studies (Tylstedt et al., 1997). The study was approved by the Local Ethics Committee (no. 99398, 22/9 1999, cont., 2003, no. C254/4, no. C45/7 2007, Dnr. 2013/190). The study adhered to the rules of the Declaration of Helsinki. Specimens were obtained from patients suffering from life-threatening petroclival meningioma undergoing transcochlear surgery. The patients/participants provided their written informed consent to participate in this study.

## Author Contributions

WL performed the immunohistochemistry for super-resolution microscopy and RNAscope. HR-A was the head of laboratory and planned the project, analyzed the images, and wrote the manuscript together with WL. Both authors approved the submitted version.

## Conflict of Interest

MED-EL Medical Electronics, R&D, GmbH, Innsbruck, Austria provided part-time salary support for one research group member WL in accordance with the contract agreement with Uppsala University, Sweden during 2018. The funder had no role in study design, data collection and analysis, decision to publish, or preparation of the manuscript. The remaining author declares that the research was conducted in the absence of any commercial or financial relationships that could be construed as a potential conflict of interest.

## Publisher’s Note

All claims expressed in this article are solely those of the authors and do not necessarily represent those of their affiliated organizations, or those of the publisher, the editors and the reviewers. Any product that may be evaluated in this article, or claim that may be made by its manufacturer, is not guaranteed or endorsed by the publisher.

## Footnotes

1
http://www.scilifelab.se

## Supplementary Material

Supplementary Video 1 3D movie visualizes *ATP1B1* mRNA transcripts in human type I spiral ganglion cells (seven slices, 545.52 nm). *ATP1B1* is restricted to neurons. Estimated number of gene puncta per cell (type I) was 360.
